# 20 million pregnant women with group B streptococcus carriage: consequences, challenges, and opportunities for prevention

**DOI:** 10.1097/MOP.0000000000001223

**Published:** 2023-02-16

**Authors:** Proma Paul, Bronner P. Gonçalves, Kirsty Le Doare, Joy E. Lawn

**Affiliations:** a Maternal, Adolescent, Reproductive & Child Health (MARCH) Centre, London School of Hygiene & Tropical Medicine; b Paediatric Infectious Diseases Research Group, Institute of Infection and Immunity, St. George's, University of London, London, UK; c Makerere University Johns Hopkins University, Kampala, Uganda

**Keywords:** group B streptococcus, IAP, maternal vaccination

## Abstract

Intrapartum antibiotic prophylaxis (IAP) is currently the only recommended preventive approach against clinical consequences of maternal Group B Streptococcus (GBS) colonization. In this review, we discuss new findings of total perinatal GBS burden and relative effectiveness of differing targeting of IAP, notably microbiology-based and risk factor-based screening, including potential limitations. Finally, we provide updates on maternal GBS vaccines and their potential cost-effectiveness in disease reduction.

**Recent findings:**

Updated estimates of the burden of GBS related to pregnancy outcomes show (1) early-onset GBS disease incidence and deaths are high in some low- and middle-income countries where IAP has not been implemented and (2) late-onset GBS disease, preterm birth, and stillbirth, which are not preventable by IAP, remain a public health problem in both high and low-middle income settings. Observational evidence indicates that microbiology-based screening may be more effective than risk factor-based screening, but even in high-income countries, compliance is imperfect. To address the need for alternative prevention strategies, several maternal vaccine candidates are in clinical development, and modelling suggests these could be cost-effective in most scenarios.

**Summary:**

Recent progress in GBS vaccine research holds promise of reducing the large and preventable burden of mortality and disability caused by GBS disease, especially in higher-burden settings where clinical and laboratory services may be limited. Importantly vaccines also hold potential to prevent GBS stillbirths and GBS-associated preterm births.

## INTRODUCTION

For decades now, it is known that Group B streptococcus (GBS) is a leading cause of neonatal infection in high-income countries and is associated with frequent morbidity and mortality. Recent estimates suggest that GBS infection is in fact a global problem, with a large fraction of the associated burden in low- and middle-income countries [1^▪▪^]. Indeed, it was estimated that ∼20 million pregnant women worldwide were colonised by GBS in 2020 and almost 400,000 children presented with either early-onset GBS (EOGBS, presenting 0-6 days after birth) or late-onset GBS (LOGBS, presenting days 7–89 after birth). Furthermore, over 90,000 infant deaths were estimated to occur, nearly half of which in Sub-Saharan Africa. 

**Box 1:**
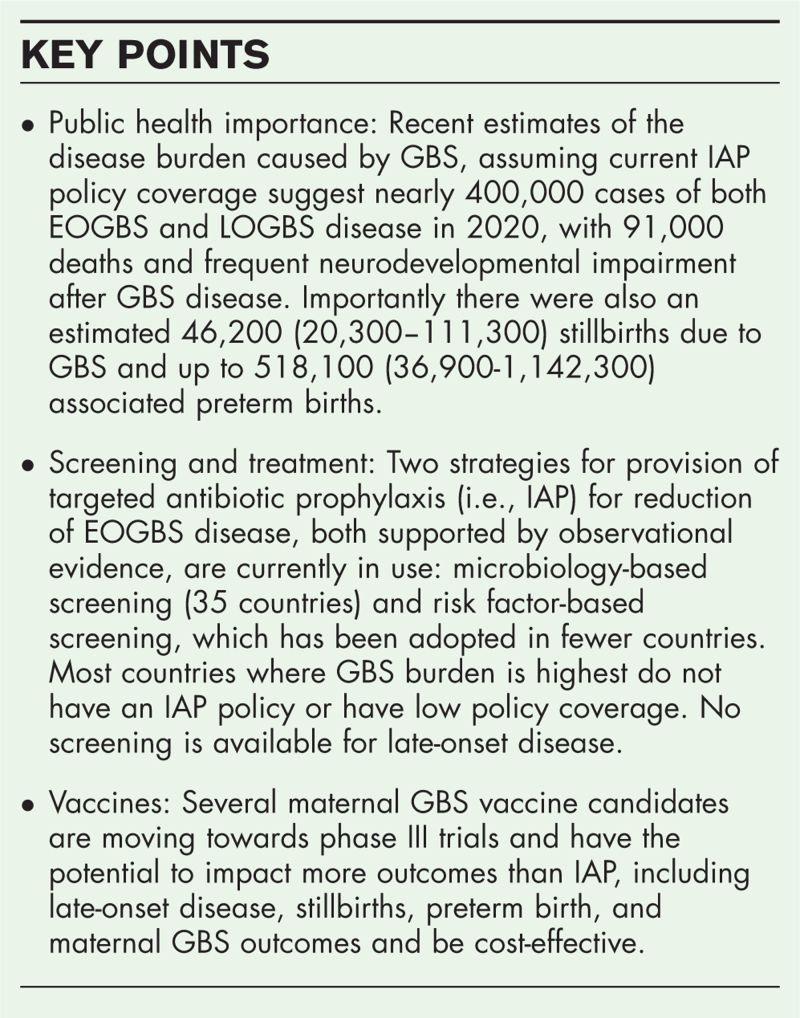
no caption available

**FIGURE 1:**
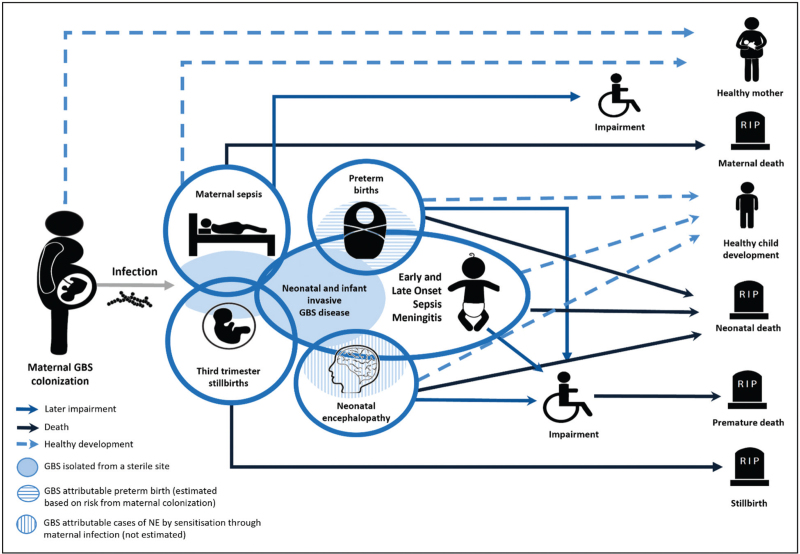
Disease scheme for outcomes of Group B Streptococcus (GBS). The blue solid line arrows indicate later impairment, the black solid line arrows indicate death, and the blue dashed line arrows indicate healthy development. Figure adapted from *Lawn JE et al.* Clinical Infectious Diseases 2017 (2).

**FIGURE 2:**
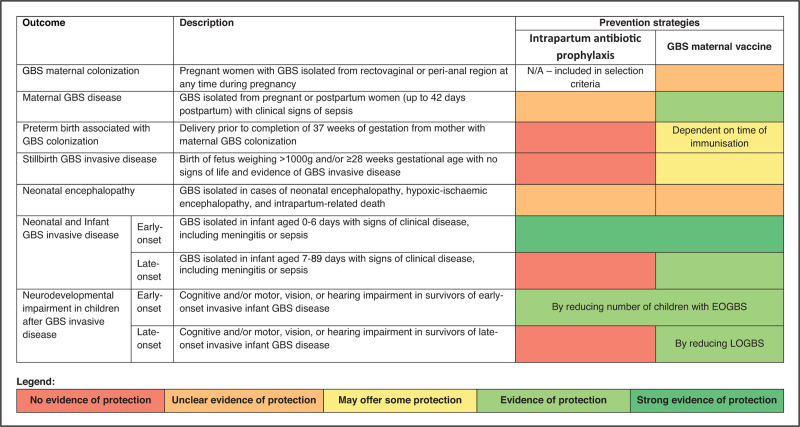
Definitions for GBS outcomes and the potential pathways of prevention comparing intrapartum antibiotic prophylaxis and maternal vaccines. Red indicates no evidence of protection, orange indicates unclear evidence of protection, yellow indicates evidence that it may offer some protection, light green indicates evidence of protection and dark green indicates strong evidence of protection. Definitions adapted from *Lawn JE et al.* Clinical Infectious Diseases 2017 (2).

Much of the clinical research on perinatal GBS infection has been on infant invasive disease. However, this pathogen is also associated with other health outcomes [2] (Fig. 1), including as a causative factor for stillbirths [3], and a risk factor for preterm births [4] and for long-term sequelae in survivors of acute disease [5,6^▪▪^,^7^,^8^,9^▪^,^10^^▪▪^]. It was estimated that 46,200 (95% posterior interval 20,300–111,300) stillbirths resulted from in utero GBS infection in 2020, and up to 518,100 (36,900–1142,300) preterm births might have been associated with GBS colonisation [1^▪▪^]. The many potential negative consequences of maternal GBS colonisation imply the need for an effective prevention approach that can reduce the risk of multiple outcomes (Fig. 2).

Here, we discuss the preventive approach currently in use, intrapartum antibiotic prophylaxis (IAP), which provides protection against EOGBS. Whilst maternal GBS vaccines, that is, vaccines to be given to pregnant women to protect their neonates, are not currently available, they are under development and will also be discussed. The objectives of this review are:

(1) To review the evidence and compare two approaches to identify pregnant women to receive IAP,
(2) To update on maternal GBS vaccine development,
(3) To discuss the potential impact of these two prevention strategies (IAP and vaccine) on clinical outcomes.

## GBS SCREENING AND INTRAPARTUM ANTIBIOTIC PROPHYLAXIS

The current approach to prevent GBS disease in early infancy consists of identifying pregnant women whose newborns are at higher risk of developing invasive disease and administering intravenous antibiotics, for at least 4 h, after onset of labor. This strategy, referred to as IAP, has been adopted in 60 of 95 countries included in a recent review [11]. Two different methods are used to identify at-risk newborns: microbiology-based screening and risk factor-based screening (Table 1). In the next subsections, these approaches of IAP are described, and evidence on their comparative effectiveness, discussed.

**Table 1:** Comparison of universal microbiology-based and risk factor-based screening for GBS in pregnancy

	Microbiology-based screening	Risk factor-based screening
Resources	Infrastructure to perform the assay, which will vary depending on culture method or PCR-based method. For intrapartum testing, a 24-h infrastructure needs to be in place. ASM recommends enrichment broth for both methods.	For assessment of presence of most risk factors, GBS testing is not required; note however that in settings where history of GBS detection is part of screening, testing infrastructure would have been necessary.
Timing	Culture based: depends on method, but usually 24–48 h Molecular biology based: < 2 h if used without enrichment broth step (it can be used for intrapartum screening)	No time delays as testing not required.
Selection of pregnant women for IAP	Based on diagnostic testing. In many countries where this screening approach has been adopted, some risk factors are used in combination with microbiology-based screening to define the population that should receive IAP	Based on the presence of risk factors.
Antibiotic treatment	Penicillin, Ampicillin First Generation Cephalosporin or Clindamicyn if penicillin allergy Vancomycin if penicillin allergy and clindamycin resistance	
Resistance considerations	There is only limited evidence of Penicillin resistance in GBS isolates [48–50]. On the other hand, resistance to Clindamycin is frequent in some settings (e.g., 20.8% in the US [20])	

### Microbiology-based screening to identify at-risk newborns

Maternal colonisation with GBS is necessary for the development of invasive disease in the first days after birth, and, consistent with this, epidemiological studies have estimated strong associations between GBS carriage during pregnancy and neonatal disease (e.g., unadjusted odds ratio 17.7 95% confidence interval 1.9–163.5 in [12]). This aspect of the pathogenesis motivates the use of bacterial colonisation testing to identify pregnant women who should receive antibiotics to reduce GBS transmission to neonates.

This screening strategy has been adopted as part of IAP policy by at least 35 countries [11], primarily high-income, and involves collection of rectal and vaginal swabs for GBS detection. Until recently, in the US, the gestational age window for GBS screening was 35 to 37 weeks based on guidance by the Centers for Disease Control and Prevention; this has recently changed and the current recommendation from the American College of Obstetricians and Gynecologists is for microbiological sampling to occur between weeks 36-0/7 and 37-6/7 of gestational age [13^▪^]. There is variation between countries both in terms of timing and anatomical location of swab sampling [11].

Culture is the most used diagnostic method, with selective enrichment broth medium having higher sensitivity compared to other culture-based methods (see [14^▪^] for recommendations on sample collection, transport, and detection). Other approaches, however, have promise, including polymerase chain reaction (PCR)-based methods; indeed, recent studies, in various settings, suggest molecular biology methods are sensitive, particularly when a step with selective enrichment broth is used [14^▪^].

An underlying assumption of the microbiology-based approach is that GBS colonisation at screening time is a good predictor of colonisation at delivery. Three challenges affect this strategy: (1) some GBS carriers will clear their infections by delivery, which implies they will receive antibiotics despite their newborns not being necessarily at higher risk of disease; (2) a fraction of pregnant women who are GBS-colonised at screening might have false negative culture results; (3) some pregnant women who were truly uninfected at screening become colonised by delivery.

Evidence for these different challenges has been reported in various settings. For example, a recent French study suggested ∼40% of pregnant women with evidence of GBS colonisation at 35 to 37 gestational weeks were not colonised at delivery [15] and in 18 US clinical centers, 53% of infants with EOGBS sepsis were born to women with negative GBS screening [16]. Sensitive diagnostic methods with a shorter time-to-result could be used for intrapartum screening, and potentially reduce these problems. PCR-based methods have been assessed in clinical studies (15–17) and their future wider use will likely depend on associated costs [17].

Despite these difficulties in identifying GBS-colonised pregnant women, a recent meta-analysis of observational data estimated microbiology-based screening reduced EOGBS risk by approximately 70% and 60% compared to no preventive policy or risk factor-based approach, respectively [18^▪^]. However, some studies in that meta-analysis used historical, rather than concurrently recruited, comparator cohorts. In one large study (*N* = 5144), performed in the US, microbiology-based screening resulted in a lower risk for EOGBS (adjusted relative risk 0.46 95% confidence interval 0.36 – 0.60; reference, risk factor-based screening) [19]. Studies that assessed population-level incidence over long periods of time provide additional observational evidence of the effect of this strategy. For example, in the US [20], the incidence went from 0.37 per 1000 live births in 2006 to 0.23 in 2015; a similar pattern was observed in France [21]. However, this approach of IAP, as well as the risk factor-based approach discussed below, does not affect LOGBS incidence to the same degree or at all [21,22]

Although microbiology-based screening and IAP is an effective approach against infancy GBS disease, several factors complicate its implementation and even in countries where diagnostic methods are widely available, compliance is not perfect. In a study from the US, 85% of pregnant women were screened for GBS colonisation, only half of them were known to be tested at week 35 or later of gestation, and of those who tested positive for GBS carriage 87% received antibiotics [23]. Two recent analyses of American data reported that 21.8% [20] and 37.5% [16] of EOGBS cases did not receive IAP despite indication. Evidence of inadequate policy compliance has also been reported in other high-income countries [24,25]. If coverage is improved, additional reductions in EOGBS incidence should be possible, but may be increasingly expensive per case treated.

### Risk factor-based screening

The other approach to identify at-risk newborns is based on known risk factors for GBS neonatal infection, including fever, preterm delivery, GBS bacteriuria, prolonged rupture of membranes, and a previous child with EOGBS [11]. The advantage of this strategy compared to microbiology-based screening relates to its lower cost, as no diagnostic tests are performed, and to the potentially lower number of antibiotic doses administered, although similar frequencies of antibiotic use have been reported with the two screening strategies [19].

Fewer countries currently use this approach, including the UK, Denmark, and the Netherlands, among others. There is evidence of EOGBS incidence reduction in some countries (e.g., Sweden [26], Denmark [27], and New Zealand [28]). However, in the Netherlands there was an increase in incidence between 1987 and 2011 (Dutch screening guidelines introduced in 1999) [29], possibly related to the expansion of GBS virulent lineages [30]. A contributing explanation for the limited effectiveness of this approach is the large fraction (∼40%) of early-onset cases in newborns of mothers who do not present risk factors [26,28]. However, this evidence is primarily observational. A large clinical trial currently underway in the UK aims to compare microbiological-based screening using culture at 35 to 37 weeks, intrapartum PCR testing at start of labor, and risk factor-based prevention [31]. As with microbiology-based screening, issues of coverage and compliance are also present in this type of screening.

## MATERNAL GBS VACCINE CANDIDATES

Although microbiology-based screening with IAP reduces EOGBS incidence, additional approaches are needed to prevent LOGBS, GBS-associated stillbirth, preterm births, and maternal infections [2]. GBS vaccines are promising. Milestones set in the Defeating Meningitis Roadmap call for licensure and WHO-prequalification of an affordable maternal GBS vaccine by 2026 and vaccine introduction in at least ten countries by 2030 [32^▪▪^]. As with immunisation with tetanus toxoid, pertussis, and influenza, the protective mechanism of maternal GBS vaccines would involve placental transfer of GBS antibodies to ensure protection when risk of invasive disease is highest [33].

Two vaccine development approaches have been prioritised: vaccines based on capsular polysaccharides and protein-based vaccines. Multivalent capsular polysaccharide vaccines include candidates in various stages of development (Table 2). As six GBS serotypes (Ia, Ib, II, III, IV, V) account for most disease burden [34], Pfizer designed a hexavalent vaccine (GBS6) to target these serotypes; Phase 2 evaluation to assess the safety and immunogenicity in pregnant women is currently underway (NCT03765073) and Phase 3 trials are set to start in 2023 [35]. Another candidate, developed by PATH, a global public health non-profit organization, and Inventprise, a vaccine manufacturer, to address the need for low-cost vaccines, is a multivalent vaccine currently in Phase 1 development [36].

**Table 2:** GBS vaccine candidates in the development pathway

Vaccine candidate	Serotype target	Preclinical	Phase 1	Phase 2	Trials in Pregnant Women	Phase 3	Trial locations
Polysaccharide conjugate vaccines							
Monovalent and bivalent conjugates (TT / CRM197 CPS)	*TT monovalent:* Ia, Ib, II, III, IV^a^, V, VI^a^, VII^a^, VIII^a^ *TT bivalent:* II, III *CRM197 monovalent:* V	√	√	√	√		No longer in development
Trivalent CRM197-CPS conjugates	Ia, Ib, III	√	√	√	√		No longer in development
Pentavalent TT CPS conjugates	TBC	√	√				TBC
Hexavalent CRM197-CPS conjugates	Ia, Ib, II, III, IV, V	√	√	√	√	√^b^	South Africa, UK, US, Uganda
Biotinylated CPS conjugates		√					
Protein-based vaccines							
N-terminal domains of the Rib and AlphaC proteins	N/A	√	√	√	√	√^b^	Denmark, South Africa, Uganda, UK
Pilus proteins		√					
Other proteins		√					

a Only in preclinical trials.

b Planned for 2023. TBC, to be confirmed.

Protein subunit vaccines could potentially cover more GBS strains and address concerns surrounding potential serotype replacement, as described for capsular polysaccharide pneumococcal vaccines [37], or capsular switching, which has been observed with natural GBS infection [38]. While several protein-based vaccines are in preclinical development, one candidate, developed by Minervax, targeting the N-terminal domain of the family of alpha-like surface proteins (GBS-NN) was found safe and immunogenic in nonpregnant women (Table 2), and is now in Phase 2 trials to evaluate immunogenicity and safety in pregnant women (NCT04596878, NCT05154578).

In 2022, the vaccine candidates GBS6 and GBS-NN were awarded PRIME status by the European Medicines Agency, which means enhanced support for the development as GBS vaccines are considered an unmet need [39–41]. Phase 3 trials are expected to start for both vaccines in 2023 [35,41].

Complexities in developing pregnancy vaccines, misconceptions regarding the success of IAP, and key data gaps in the global burden have contributed to the sluggish advancement of GBS vaccines [42]. As vaccine development progresses, a potential obstacle to licensure is the sample size needed in a Phase 3 trial to assess efficacy against invasive disease. Indeed, it has been estimated that such a trial would require 30,000–1800,000 participants, depending on vaccine efficacy and disease incidence, and would therefore require significant time and resources [37]. A recent review summarised the potential paths to licensure for GBS vaccines [43^▪▪^]. Compared to the conventional approval pathway, an accelerated approval pathway by regulatory authorities, such as the US Food and Drug Administration or the Medicines and Healthcare Products Regulatory Agency, might lead to licensure of a GBS vaccine based on a surrogate immunological endpoint and use in pregnant women. Such an approach was favourably discussed during the Vaccine and Related Biological Products Advisory Committee in May 2018 and could allow licensure based on serocorrelate of protection followed by a confirmatory effectiveness study postlicensure. Alternatively, an immunological endpoint could be nested in a trial with a clinical endpoint, where trial size would be based on the clinical endpoint, and immunogenicity results could be used for accelerated approval, before efficacy estimates based on disease become available [43^▪▪^]. Both approaches rely on validated serological correlates of protection using standardised assays. For this reason, a consortium was established to develop standardised assays and reagents. The results of these interlaboratory studies have shown good agreement between laboratories using standard reference reagents and protocols [44]. Serocorrelates of protection studies using natural immune sera are currently underway in the UK, USA, South Africa, and in the PREPARE consortium (Malawi, Uganda, Netherlands, France, Italy) to establish the concentration of antibodies associated with a risk reduction threshold for invasive disease [45].

## POTENTIAL IMPACT OF PREVENTION STRATEGIES

IAP prevents the outcome with peak incidence immediately after birth, early-onset disease. However, even in Europe and North America, where policy coverage is highest, thousands of EOGBS cases still occur every year. Equally importantly, risk of LOGBS, which more often presents as meningitis, is not reduced by IAP. Given the risk of moderate/severe neurodevelopmental impairment after GBS meningitis, estimated at 20.7% (95% posterior interval 16.1–25.6) [1^▪▪^], it is possible that even where IAP has reduced EOGBS, considerable burden will persist due to acute LOGBS and the associated long-term sequelae. Other outcomes, including GBS-associated preterm births and stillbirths are not likely to be impacted by IAP, although surveillance data from the US suggest reduced incidence of maternal disease after IAP implementation [22].

A maternal GBS vaccine on the other hand could potentially prevent both EOGBS and LOGBS. Indeed, according to an estimation in the *Group B streptococcus Full Value of Vaccine Assessment*[46^▪▪^], ∼87,000 late-onset cases and ∼127,000 early-onset cases estimated to occur annually under current IAP coverage could be prevented if a vaccine with 80% efficacy were deployed globally, and as a consequence, ∼20,000 moderate/severe impairment cases would be averted. Although questions remain on how GBS colonisation increase risk of prematurity or lead to stillbirth, if maternal vaccines enhance GBS clearance, or prevent colonisation in pregnancy, vaccination could prevent these outcomes. It has been estimated that a vaccine with 80% efficacy could potentially prevent 23,000 GBS-related stillbirths.

Maternal GBS vaccine use could also have the additional benefit of reducing antibiotic administration for IAP and treatment of vaccine-prevented early-onset and late-onset cases. If we assumed two scenarios – one in which microbiology-based screening using culture rather than PCR-based intrapartum testing [47] would eventually be adopted universally in the absence of an effective vaccine, and a second scenario where, with deployment of a vaccine, IAP would no longer be in use; in the second scenario up to ∼20 million pregnant women would not need to receive IAP every year. In reality, both scenarios are unlikely for different reasons: (1) adoption of microbiology-based screening is not feasible in many settings in the near-future, (2) in many countries a large fraction of births still happens at home, which precludes intrapartum antibiotics; (3) it is unclear whether vaccine availability would lead to discontinuation of IAP policy. For example, it is conceivable that for newborns at high risk of disease, for example, preterm infants, IAP would still be beneficial in further reducing risk. GBS vaccine deployment would likely reduce the global number of IAP- and disease treatment-related antibiotic doses administered, potentially slowing spread of antimicrobial resistance, and preventing possible, but still under-researched consequences to the microbiome.

## CONCLUSION

For the last three decades, IAP has saved lives of children who may otherwise have developed EOGBS in some higher income settings, and this strategy could be improved by better policy coverage and by improvements in diagnostic methods. However, common and serious clinical consequences of maternal GBS colonisation (notably LOGBS, stillbirths, and preterm birth) are not prevented by IAP. Maternal vaccines, which may potentially provide protection against the different presentations of this infection and are feasible to scale, could have a major impact on disease burden, in particular in limited resourced settings, where incidence is high, and IAP is challenging to implement. A recently published WHO report [46^▪▪^], which calls for the development of maternal GBS vaccines to reduce GBS burden, has increased momentum for development of GBS vaccine candidates, many of which have been stuck in the pipeline for decades.

## Acknowledgements

*We are grateful to the following research groups, with whom we have collaborated on GBS research epidemiological studies: the GBS Danish and Dutch collaborative group for long-term outcomes, and the GBS low-income and middle-income country collaborative group for long-term outcomes. We also would like to thank members of the WHO Scientific Advisory Group, ISSAD Committee (issad.org), Ajoke Sobanjo-ter Meulen, and Kate Fay.*

### Financial support and sponsorship

*This work was supported by a grant (INV-00908) from the Bill & Melinda Gates Foundation to the London School of Hygiene & Tropical Medicine (PI, Joy Lawn). The funders had no role in study design, data collection and analysis, decision to publish, or preparation of the manuscript.*

### Conflicts of interest

*There are no conflicts of interest.*

## REFERENCES AND RECOMMENDED READING

Papers of particular interest, published within the annual period of review, have been highlighted as:

▪ of special interest
▪▪ of outstanding interest
